# Pioglitazone Improves Cognitive Function via Increasing Insulin Sensitivity and Strengthening Antioxidant Defense System in Fructose-Drinking Insulin Resistance Rats

**DOI:** 10.1371/journal.pone.0059313

**Published:** 2013-03-20

**Authors:** Qing-Qing Yin, Jin-Jing Pei, Song Xu, Ding-Zhen Luo, Si-Qing Dong, Meng-Han Sun, Li You, Zhi-Jian Sun, Xue-Ping Liu

**Affiliations:** 1 Department of Senile Neurology, Provincial Hospital Affiliated to Shandong University, Jinan, Shandong, P. R. China; 2 Department of KI-Alzheimer Disease Research Center, Karolinska Institutet, Stockholm, Sweden; 3 Department of Anti-Ageing, Provincial Hospital Affiliated to Shandong University, Jinan, Shandong, P. R. China; 4 Department of Central Lab, Provincial Hospital Affiliated to Shandong University, Jinan, Shandong, P. R. China; University of Pittsburgh, United States of America

## Abstract

Insulin resistance (IR) links Alzheimer’s disease (AD) with oxidative damage, cholinergic deficit, and cognitive impairment. Peroxisome proliferator-activated receptor γ (PPARγ) agonist pioglitazone previously used to treat type 2 diabetes mellitus (T2DM) has also been demonstrated to be effective in anti-inflammatory reaction and anti-oxidative stress in the animal models of AD and other neuroinflammatory diseases. Here, we investigated the effect of pioglitazone on learning and memory impairment and the molecular events that may cause it in fructose-drinking insulin resistance rats. We found that long-term fructose-drinking causes insulin resistance, oxidative stress, down-regulated activity of cholinergic system, and cognitive deficit, which could be ameliorated by pioglitazone administration. The results from the present study provide experimental evidence for using pioglitazone in the treatment of brain damage caused by insulin resistance.

## Introduction

Insulin resistance is one of the core defects in type 2 diabetes mellitus (T2DM) and this defect leads to hyperinsulinemia that compensates for the reduced efficacy of insulin in peripheral tissues. Insulin resistance may be manifested only by mild glucose intolerance for many years prior to the onset of frank diabetes, as the pancreas is able to generate sufficient levels of insulin to maintain glucose levels beneath the diabetic threshold. About 60–70% of T2DM patients have diabetic neuropathy in peripheral and central nervous systems (CNS) [1]. In CNS, deterioration of cognitive function such as learning and memory impairment has been proven to be associated with insulin resistance [2]–[4].

Oxidative damage plays an important role in the pathogenesis of T2DM, Alzheimer’ disease (AD) and other neurological diseases [5], [6]. Oxidative stress arises due to the imbalance of the production of free radicals and cellular antioxidant defense mechanism. The excessive production of free radicals in brain that has insulin resistance may attack many cellular components including membrane lipids and proteins, resulting in neuronal damage and dysfunction [7], [8]. On the other hand, the antioxidant enzyme activities such as glutathione peroxidase (GPx), catalase (CAT), superoxide dismutase (SOD) as well as non-enzymatic antioxidants including reduced glutathione (GSH) were reduced in the brains of insulin resistance rats [9]–[13], suggesting that a declined antioxidant ability is induced by insulin resistance. Accumulating evidences have demonstrated the link between free radical and neuronal degeneration, which highlights the importance of antioxidants in the treatment of neurodegenerative disorders including diabetes-associated cognitive decline [5]. It is thought that oxidative damage contributes to learning and memory impairments in rat models with insulin resistance [7], [14].

The deficit of cholinergic neurotransmission is an important mechanism in the pathogenesis of AD, and it correlates closely with the severity of cognitive impairment in AD patients, as well as in humans, rats, and mice with central insulin resistance [7], . The concentration of acetylcholine (Ach), the key neurotransmitter involved in cognitive process, is influenced by acetylcholinesterase (AChE) and choline acetrltransferase (ChAT) activities [16]. ACh is increased by inhibiting the activity of AChE or promoting the activity of ChAT, by which the impairment of cognitive function could be ameliorated [15], [16]. AChE is responsible for degradation of ACh to acetate and choline in synaptic cleft [17]. Cholinergic replacement strategy is the only practiced treatment used for AD patients today. The mechanisms underlying the cholinergic deficit induced by insulin resistance in brain are still needed to be clarified.

Pioglitazone, a thiazolidinedione derivative, is a highly selective peroxisome proliferator-activated receptors (PPARγ) agonist. It is currently approved in the treatment of T2DM associated with insulin resistance. PPARγ agonists are known to improve insulin sensitization, modulate glucose and lipid metabolism [18], was also considered as candidate drug in the treatment of neurodegenerative disorders such as AD, Huntington’s disease, and Parkinson’s disease [19]–[21]. Besides the antioxidant ability, previous studies have shown that pioglitazone is beneficial to central cholinergic system, and ameliorates cognitive impairment in experimental dementia rats [22], [23]. In our previous studies, we found that pioglitazone partly reversed the accumulation of β-amyloid and the activation of advanced glycosylation end products (AGEs)**/**receptors for AGEs (RAGE) system in brains of fructose-drinking insulin resistance rats [24], [25]. So far, the precise molecular mechanisms as to how insulin resistance is induced by high fructose intake, resulting in cognitive impairment, and how the learning and memory deficit induced by high fructose intake is ameliorated by pioglitazone have not been completely understood.

In the present study, we tested if long-term fructose intake can induce insulin resistance, oxidative damage, and deficit in cholinergic function, and impairment in learning and memory ability in rats. We also investigated the potential therapeutic role of pioglitazone in these molecular events.

## Materials and Methods

### Animals and Grouping

Male Wistar rats, six-week-old, were obtained from the Experimental Animal Center of Shandong university, Jinan, China, and housed (3 rats/cage) at 22±2°C under diurnal cycle (light-dark: 08∶00–20∶00). The rats were given food and water ad libitum. The animals were cared in accordance with the Provisions and General Recommendation of Chinese Experimental Animals Administration Legislation. The experiment was approved by the Provincial Hospital Council on Animal Care Committee, Shandong University, China.

After adaptation for a week, the rats were randomly divided into 4 groups (10 in each group): a control group (control) and a control treatment group (Pioglitazone); a fructose group (fructose) and a fructose treatment group (Pioglitazone+fructose). Control rats were fed with plain water. Experimental rats received 10% fructose solution in drinking water for 16 weeks to develop insulin resistance. 10 mg/kg Pioglitazone (Actos™, Takeda Pharmaceuticals, Osaka, Japan) suspended in normal water was given to rats daily at a volume of 2 ml/kg/day by gavage for the last 12 weeks of the 16-week treatment period. Non-treated rats were administered with normal water at the same volume for the last 12 weeks [24].

### Reagents

Immunoreagent kit used for fasting insulin (FINS) levels was from Beijing Furui Biotechnology (Beijing, China). The assay kit for total antioxidant capability (T-AOC) was from Nanjing Jiancheng Bioengineering Institute (Nanjing, China). All other chemicals for biochemical analysis were purchased from the Sigma-Aldrich (MO, USA) unless otherwise indicated.

### Preparation of Homogenates or Supernatants

A total of 5 rats (one in control treatment group, two in fructose group, and two in fructose treatment group) were excluded from the study because they were dead or severely ill before the beginning of Morris water maze test, all other rats were killed by decapitation under anesthesia after behavioral experiments. The brains were immediately removed from each rat and washed with ice-cold normal saline, and the hippocampus and cerebral cortex were dissected. The hippocampus and cerebral cortex of the left hemisphere were weighed and homogenized in a 50 mM phosphate buffered saline (PBS) pH 7.0 containing 0.1 mmol/L ethylenediaminetetraacetic acid (EDTA). The half volume of homogenate was centrifuged at 1500×*g* for 15 min at 4°C to prepare supernatants for T-AOC, SOD, CAT, GPx, GSH, thibarbituric acid reactive substances (TABRS), ACh, and AChE assays. The other half of homogenate was further centrifuged at 12,000×*g* for 10 min at 4°C, and was prepared for protein carbonyl content (PCC) assay [26]. The hippocampus and cerebral cortex in the right hemisphere were weighed and homogenized in 50 mM PBS at PH 7.0 (10%, w/v) and centrifuged at 11,000×*g* for 15 min at 4°C for reactive oxygen species (ROS) assay [27].

### Protein Concentration Measurement

The protein concentration in homogenates or supernatants prepared above was measured by the method of Lowry et al [28], using bovine serum albumin (BSA) as standard.

### Detection of Fasting Glucose and Insulin (FINS) Levels in Plasma

Insulin sensitivity was assessed at the end of experiments by measuring insulin resistance index (IRI). The rats after behavior tests were fasted for 12 h, 3 ml blood samples collected from abdominal aorta for each case 2 days before animals were killed were centrifuged at 1200×*g* for detection of fasting blood glucose (FBG) and FINS levels. The FBG levels were determined by a glucose-oxidase biochemistry analyzer and FINS levels were measured by homogeneous phase competitive immunoradiometric assay with Immunoreagent kit using GC-911γ immunoradiometric counter (Enterprises Group of USTC, Hefei, China). IRI was calculated as formula: IRI = FBG×FINS/22.5 [24], [29].

### ROS Determination

ROS levels were quantified by 2′-7′-dichlorofluorescein-diacetate (DCFH-DA) assay as previously described [27]. Briefly, 40 µl supernatant (4.5 mg/ml), 20 µl 100 µM DCFH-DA, and 140 µl 50 mM PBS (pH 7.0) were mixed, and the mixture was incubated for 30 min at 37°C. The reaction was stopped by placing it on ice, and the formation of oxidized fluorescent 2′-7′-dichlorofluorescein (DCF) was measured by fluorimeter (F96S; Lengguang, Shanghai, China) using excitation and emission wavelengths at 488 and 525 nm, respectively. The final results were corrected for protein concentration and then expressed as percentage of the corresponding values in control group. All steps were performed in the dark and DCF formation was also monitored immediately after DCFH-DA was added to the homogenate (*t* = 0 min) in order to subtract background autofluorescence.

### TBARS Assay

The levels of lipid peroxidation were quantified by TBARS assay according to previously described [30]. 50 µl supernatant (8.5 mg/ml) was mixed with 1 ml 50 mM PBS (PH 7.0), 10 µl 0.375% thiobarbituric acid (TBA), 10 µl 15% trichloro acetic acid (TCA), 5 µl 0.25 M HCl, and 5 µl 6.8 mM 2,6-di-tert-butyl-4-methylphenol (BHT), was incubated at 95°C for 1 h. After cooling with tap water, the reaction was terminated with a mixture of 1.0 ml distilled water and 3.0 ml butyl alcohol and pyridine (15∶1, v/v). The mixture was centrifuged at 2000×*g* for 10 min. The absorbance of the supernatant was determined at 532 nm by using 1,1,3,3-teraethoxypropane (TEP) as standard. The lipid peroxidation was expressed as TBARS in nanomoles per mg protein.

### Protein Carbonyl Content Measurement

Protein carbonyl content (PCC), a marker of oxidized proteins, was measured spectrophotometrically [31]. Briefly, 300 µl mixture was prepared by adding 50 µl 5% streptomycin sulfate solution (w/v), 200 µl supernatant (9.5 mg/ml), and 50 µl 50 mM PBS (pH 7.0), and incubated for 15 min at 37°C. After centrifuged at 10,000 ×*g* for 10 min at 4°C, the pellet was dissolved in 100 µl 10 mM 2, 4-dinitrophenylhidrazine (DNPH) solved in 200 µl 2.5 M HCl. The re-suspended mixture was incubated in the dark for 1 h at 37°C. The proteins were precipitated by adding equal volume of 20% TCA (w/v), and subsequently washed three times with ethanol: ethyl acetate (1∶1, v/v). The final protein pellet was dissolved in 400 µl of 6 M guanidine hydrochloride and the absorbance was read at 370 nm. The results were calculated as nM carbonyls groups mg of protein^−1^, using the extinction coefficient of 22,000 M/cm for aliphatic hydrazones.

### SOD Assay

SOD activity was measured basing on the method of Kono [32]. Stocking solution A was prepared by mixing 10 ml of 0.001 N NaOH comtaining 5 µM xanthine with 100 ml of 1 mM PBS (PH 7.0), containing 0.1 mM EDTA, and 2 µM cytochrome c. Stocking solution B (150 µl) was prepared by mixing 100 µl of 0.2 U xanthine oxidase/ml, and 50 µl of 0.1 mM EDTA. 50 µl supernatant (8.5 mg/ml) was mixed with 2.9 ml solution A and 50 µl solution B, the mixture was incubated for 30 min at 37°C. Reaction was stopped by adding 0.2 ml of 16% acetic acid (v/v) containing 2.6 mM sulfanilic and 38.6 µM naphthyl ethylenediamine. The absorbance at 550 nm was monitored. A blank was run by substituting 50 µl ultra pure water for the supernatant. SOD activity was expressed as units per milligram of protein.

### CAT Assay

CAT activity was measured by the method of Goth [33]. Briefly, 50 µl supernatant (8.5 mg/ml) was mixed with 50 µl of 6.5 µM hydrogen peroxide as substrate, and 100 µl of 50 mM PBS (PH 7.0), and incubated for 60 s at 37°C. The mixture was added with 100 µl of 32.4 mM ammonium molybdate to terminate the reaction. The absorbance was measured at 405 nm. One unit of the enzyme was defined as milli-moles of hydrogen peroxide degraded per min per mg protein.

### GPx Assay

GPx activity was estimated according to the method of Hafemen [34]. Briefly, the 300 µl mixture contained 40 µl supernatant (8.5 mg/ml), 30 µl 3 µM GSH, 30 µl 6.5 µM hydrogen peroxide, and 200 µl 50 mM PBS (pH 7.0) was prepared, and incubated at 37°C for 3 min, followed by precipitating proteins with 100 µl 10% TCA (v/v). The resulting supernatant was collected and the reaction was stopped with 100 µl of 20 mM disodium hydrogen phosphate and 100 µl 0.025 mM 5, 5,-dithiobis (2-nitro-benzoic acid) (DTNB). The absorbance was measured at 412 nm. The unit of GPx activity was expressed as micromoles GSH oxidation per min per mg protein.

### GSH Assay

GSH level was measured according to a previously described method [35]. 100 µl supernatant (8.5 mg/ml) was filled with 700 µl 0.1 mM PBS (pH 7.0), 100 µl 0.6 mM DTNB and 80 µl 0.2 mg/ml nicotinamide adenine dinucleotide phosphate (NADPH) to make a total volume of 980 µl, and the mixture was incubated at room temperature for 5 min. After mixing, glutathione reductase (20 U/ml, 20 µl) was added to stop the reaction. The formation of 5-thio-2-nitrobenzoic acid parallels the absorbance at 412 nm. GSH levels were expressed as µg per mg protein.

### T-AOC Assay

The T-AOC was determined according to method described by Benzie and Strain. [36]. This method allows designing “total” reducing power of the electron donating antioxidants present in examined samples. In reaction mixture ions Fe^3+^ are reduced to Fe^2+^ and blue complex Fe^2+^-TPTZ (2,4,6-Tri(2-pyridyl)-*s*-triazine) is produced. 20 µl supernatant (8.5 mg/ml) was added to 2.25 ml reaction buffer containing 130 mM acetate PH 3.6, 4.2 mM TPTZ prepared in 19 mM HCl, and 8.5 mM FeCl_3_·6H_2_O), and incubated at room temperature for 5 min, then the reaction and the absorbance for reaction mixture was read at 593 nm immediately and 10 min after. Final readings were based on standard curve consisting of five to eight standard points, covering the entire range of expected concentrations which was prepared with aqueous solutions of known Fe^2+^ concentration (range 100–1000 µmol/ml). Data were expressed as U/mg protein.

### AChE Assay

AChE activity was measured according to a previously described method [17]. 670 µl reaction mixture containing 33 µl supernatant (8.5 mg/ml), 470 µl 1 mM PBS (pH 8.0), and 167 µl 2% DTNB was incubated for 5 min at 37°C. Then, 280 µl 2 mM acetylcholine iodide were added to the mixture. After incubation for 5 min at 37°C, the reaction was terminated by adding 50 µl of 4 mM neostigmine. The absorbance was read at 412 nm at room temperature. 1 U AChE activity was defined as the number of hydrolyzed micromoles of acetylthiocholine iodide per min per microgram of protein. AChE activity was expressed as units per milligram of protein.

### ACh Assay

The ACh content was estimated by the method of Yang [37]. Briefly, a total of 4.1 ml reaction mixture containing 0.8 ml (8.5 mg/ml) supernatant, 1 ml 2 M calabarine sulfate, 0.8 ml 1.85 M TCA, and 1.5 ml pure water was prepared, and incubated for 5 time at room temperature. After vortexing, the mixture was first centrifuged at 2000×*g* for 10 min, and the resulting supernatant was again centrifuged at 2000×*g* for 10 min. The collected supernatant (1 ml) was mixed with 2 M alkalinity hydroxylamine (1∶1) for 10 min at room temperature. The reaction is stopped by adding 0.5 ml of 4 M HCl and 0.5 ml of 0.37 M ferric chloride. The absorbance was measured at 540 nm at room temperature. ACh content were expressed as µM per mg of protein.

### Histopathological Examination

The cerebra (5 cases) were dehydrated and embedded in paraffin blocks after post-fixed with 4% paraformaldehyde. Four 10 µm coronal sections for each case taken from −4.0 mm posterior from bregma were stained with hematoxylin-eosin. Cells in the hippocampus and temporal coxtex were observed under a light microscope (Olympus, Japan) and pictures were taken at 200× magnification. The cells with a round or oval-shaped nuclei and no shrinkage or edema were regarded as undamaged.

### Experiments of Behavioral Test

The Morris water maze (MWM) test, which consisted of 5-day training (visible and invisible platform training sessions) and a probe trial on day 6, was used to evaluate the learning and memory ability of the rats [38], [39]. The maze is a circular pool (100 cm in diameter, 50 cm in height) which was painted black and filled with 22±2°C milk water to a depth of 30 cm with a platform (9 cm in diameter). It was placed in a dimly lit, sound-proof test room. The platform was placed in the center of the 4 quadrants and remained there throughout the experiment. Each rat was individually trained in both visible-platform (days 1–2) and hidden-platform (days 3–5) versions. Visible platform training was performed for baseline differences in vision and motivation; the platform was placed 1 cm below the surface of the water and was indicated by a small flag (5 cm in height). The hidden-platform trial evaluated the spatial learning and was used to determine the retention of memory to find the platform. During the training days, the platform was submerged 1 cm below the surface of the water and the flag was removed. On each day, the rat was subjected to 4 trials with a 1-h interval between trials. Each trial lasted for 90 seconds unless the animal reached the platform. The test was ended and the rat was gently placed to the platform for 30 seconds if a rat failed to find the platform within 90 seconds. On day 6, the platform was removed and the probe trial started, and rats had 90 seconds to search for the platform. The proportion of time and path that the rats had spent in the target quadrant, in which the hidden escape platform was previously located, was noted. In addition, the times of the rats swimming through the hidden escape platform were also noted by an overhead camera connected to a computerized tracking system (HVS Image, Hampton, UK).

### Statistical Analyses

All results were reported as mean ± S.E.M. Statistical significance was assessed with one-way analysis of variance (ANOVA) followed by Tukey–Kramer test for post hoc comparisons between groups, except the statistical analysis for the acquisition phase of MWM test is repeated-measures ANOVA. The significance of differences was determined using two-way ANOVAs when two factors were assessed. The acceptable level for statistical significance was *P*<0.05.

## Results

### Pioglitazone Improves Insulin Sensitivity in Fructose-drinking Insulin Resistance Rats

As seen from **Table 1**, levels of plasma insulin and insulin resistance index, but not plasma glucose, in fructose-drinking insulin resistance rats were significantly higher than those in control rats (34.6±4.5 mU/L, 8.52±0.62 in fructose-drinking insulin resistance rats and 14.5±2.9 mU/L, 3.30±0.34 in control rats, respectively, *P*<0.01). Pioglitazone treatment significantly reduced the levels of plasma insulin and insulin resistance (19.4±4.0 mU/L and 4.64±0.33, respectively, *P*<0.05 for both) compared to the level of the fructose-drinking insulin resistance rats. There were no significant difference in the levels of plasma insulin and insulin resistance index between control and pioglitazone-treated control rats (13.9±3.3 and 2.98±0.28, respectively, *P>*0.05).

**Table 1:** Effects of pioglitazone on plasma glucose and insulin in fructose-drinking insulin resistance rats.

Groups (n)	Body weight (g)	Fasting blood glucose (mmol/L)	Fasting insulin (mU/L)	Insulin resistance index
**Control (10)**	397.4±19.3	5.12±0.78	14.5±2.9	3.30±0.34
**Pioglitazone (9)**	401.5±15.9	4.83±0.39	13.9±3.3	2.98±0.28
**Fructose (8)**	409.3±12.9	5.54±0.58	34.6±4.5^a^	8.52±0.62^a^
**Pioglitazone+fructose (8)**	412.2±15.9	5.38±0.47	19.4±4.0^bc^	4.64±0.33a,c

Data are shown as mean ± S.E.M.

a *P*<0.01,

b *P*<0.01 versus control group;

c *P*<0.05 versus untreated fructose-drinking group.

### Pioglitazone Up-regulates Anti-oxidant Defense System in Fructose-drinking Insulin Resistance Rats

**Figure 1:**
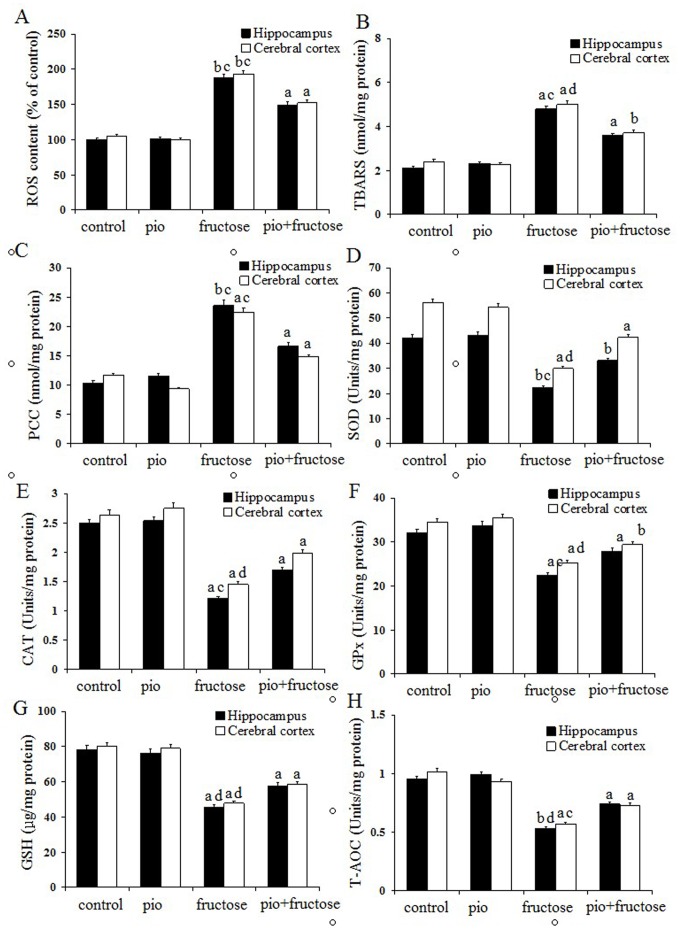
Effects of pioglitazone on the levels of ROS, TBARS, and contents of carbonyl proteins in the hippocampus and cerebral cortex of fructose-drinking insulin resistance rats (A–C); effects of pioglitazone on the activities of antioxidant enzymes (SOD, CAT and GPx) (D–F), the levels of GSH and T-AOC (G–H) in the hippocampus and cerebral cortex of fructose-drinking insulin resistance rats. The control group (control) and the control treatment group (pio: pioglitazone) were treated with normal drinking water while the fructose group (fructose) and fructose treatment group (pio+fructose: pioglitazone+fructose) were treated with 10% fructose solution for 16 weeks to develop insulin resistance. The fructose treatment group was orally administrated pioglitazone (10 mg/kg d) for the last 12 weeks of the 16-week period. Data are mean ± SEM. Data are mean ± SEM. The number of rats in groups control, pio, fructose and pio+fructose was 10, 9, 8, and 8, respectively. ^a^ *P*<0.05, ^b^ *P*<0.01 versus control group;^ c^ *P*<0.05, ^d^ *P*<0.01 versus untreated fructose-drinking group.

As shown in the panels A, B, and C of Fig. 1, ROS overproduction (oxidative stress), TBARS (lipid peroxidation), and carbonyl content (protein oxidation, PCC) were measured. Fructose-drinking insulin resistance rats showed significant higher levels of ROS (*P*<0.01, *P*<0.01), TBARS (*P*<0.05, *P*<0.05) and carbonyl (*P*<0.01, *P*<0.05) in both the hippocampus and cerebral coxtex than control rats. Reduced levels of ROS (*P*<0.05, *P*<0.05), TBARS (*P*<0.05, *P*<0.01) and carbonyl (*P*<0.05, *P*<0.05) in both hippocampus and cerebral coxtex were observed in pioglitazone (pio)-treated fructose-drinking rats compared with fructose-drinking insulin resistance rats. There were no significant differences in the levels of the three oxidative damage markers for both brain regions between control and pioglitazone-treated control rats (*P*>0.05).

**Figure 2:**
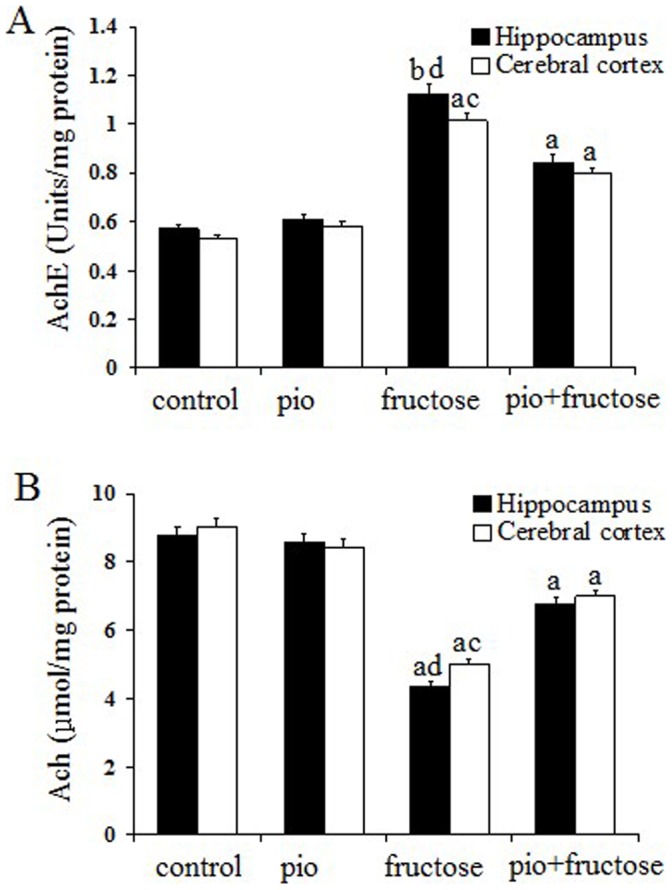
Effects of pioglitazone on AChE (A) and ACh (B) in the hippocampus and cerebral cortex of fructose-drinking insulin resistance rats. The control group (control) and the control treatment group (pio: pioglitazone) were treated with normal drinking water while the fructose group (fructose) and fructose treatment group (pio+fructose: pioglitazone+fructose) were treated with 10% fructose solution for 16 weeks to develop insulin resistance. The fructose treatment group was orally administrated pioglitazone (10 mg/kg d) for the last 12 weeks of the 16-week period. Data are mean±SEM. Data are mean±SEM. The number of rats in groups control, pio, fructose and pio+fructose was 10, 9, 8, and 8, respectively. ^a^ *P*<0.05, ^b^ *P*<0.01 versus control group;^ c^ *P*<0.05, ^d^ *P*<0.01 versus untreated fructose-drinking group.

As shown in the panels D, E, and F of Fig. 1, SOD (*P*<0.01, *P*<0.05), CAT (*P*<0.05, *P*<0.05)and GPx (*P*<0.05, *P*<0.05) activities of both hippocampus and cerebral cortex in fructose-drinking insulin resistance rats were significantly lower than those of control rats, significant increase in SOD (*P*<0.05, *P*<0.01), CAT (*P*<0.05, *P*<0.01) and GPx (*P*<0.05, *P*<0.01) activities of both brain regions in pioglitazone–treated fructose-drinking insulin resistance rats were observed as compared to fructose-drinking insulin resistance rats, and no difference in SOD, CAT and GPx activities was observed between control and pioglitazone-treated control rats (*P*>0.05). Results showed that the levels of GSH in both brain regions of fructose-drinking insulin resistance rats were significantly lower than those of control animals (*P*<0.05, *P*<0.05), and pioglitazone administration increased the levels of GSH in both brain regions (*P*<0.01, *P*<0.01) (Fig. 2G). The level of T-AOC in the hippocampus and cerebral cortex were measured to assess overall antioxidative capacity. Both brain regions demonstrated a significant decline in T-AOC in fructose-drinking insulin resistance rats (*P*<0.01, *P*<0.05), and animals in pioglitazone-treated fructose-drinking insulin resistance group revealed a significant higher T-AOC than that in fructose-drinking insulin resistance group (*P*<0.01, *P*<0.05) (Fig. 2H). No significant difference was observed between control and pioglitazone-treated control rats (*P*>0.05).

### Piogitazone Increases Cholinergic Activity in Fructose-drinking Insulin Resistance Rats

As shown in the panels A and B of Fig. 2, animals in fructose-drinking insulin resistance group showed significantly higher AChE activity in the hippocampus and cerebral cortex than control rats (*P*<0.01, *P*<0.05). Reduced AChE activity in both brain regions in pioglitazone-treated fructose-drinking insulin resistance rats were observed compared with fructose-drinking insulin resistance rats (*P*<0.01, *P*<0.05). The ACh content in hippocampus and cerebral cortex were significantly decreased in fructose-drinking insulin resistance rats compared with that in the controls (*P*<0.05, *P*<0.05). Pioglitazone administration considerably increased ACh content compared to those in the fructose-drinking insulin resistance rats (*P*<0.01, *P*<0.05), and no significant difference was found among both control group and the pioglitazone-treated control group (*P*>0.05).

### Pioglitazone cannot Ameliorate the Irreversible Brain Damage in Fructose-drinking Insulin Resistance Rats

**Figure 3:**
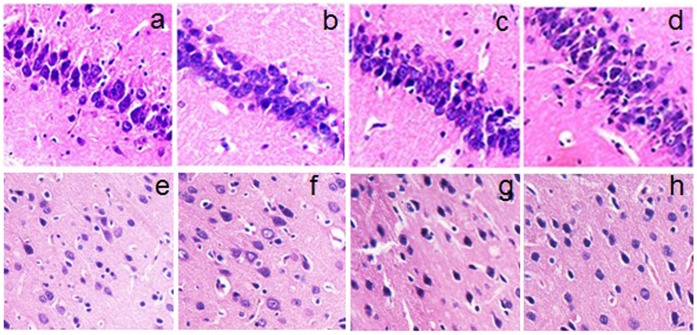
Effect of pioglitazone on histopathological changes in the hippocampus (a–d) and temporal cortex (e–h) of fructose-drinking insulin resistance rats. The hippocampal CA1 area (a,b) and temporal cortex (e,f) in the control, and the pioglitazone-treated control groups, rare damaged cells were seen. In the hippocampal CA1 area (c,d) and the temporal cortex (g,h) of the fructose-drinking insulin resistance control and the pioglitazone-treated groups, neurondegeneration was evident, but not difference was found between the two groups. The data were from five independent experiments. Magnification: ×200.

Fig. 3 is the representative photomicrograph of hematoxylin and eosin staining in the hippocampus CA1 area and temporal cortex of rats treated with fructose and pioglitazone. In the control group (a and e) and the pioglitazone-treated control group (b and f), rare damaged neurons were seen in the hippocampus and temporal cortex. However, neuronal degeneration and karyopycnosis were easily observed in the CA1 area of the hippocampus (c) and temporal cortex (g) in the fructose-drinking insulin resistance group. Pioglitazone administration showed similar pathological changes of neurons compared with the fructose-drinking insulin resistance control group.

### Pioglitazone Improves Learning and Memory Ability in Fructose-drinking Insulin Resistance Rats

**Figure 4:**
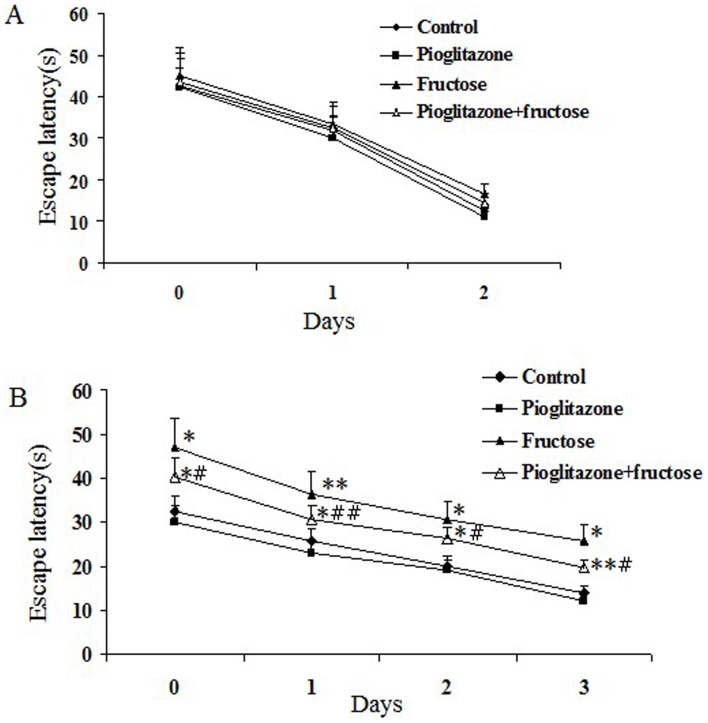
Effect of pioglitazone on memory impairment of fructose-drinking insulin resistance rats in MWM test. Day 0 represents performance on the first trial, and subsequent points indicate average of all daily trials. (A) No differences were found in the escape latency among four groups during the 2-day visible platform test. (B) Changes in escape latency to reach the hidden platform during the 3-day acquisition trials. Data are mean±SEM. The number of rats in groups control, pioglitazone, fructose and pioglitazone+fructose was 10, 9, 8, and 8, respectively. ^*^ *P*<0.05, ^**^ *P*<0.01 versus control group;^ #^ *P*<0.05, ^##^ *P*<0.01 versus untreated fructose-drinking group.

Morris water maze test was used to access the learning and memory functions in each group. As can be seen from Fig. 4A, rats in each group exhibited a similar escape latency in 2-day visible-platform test, suggesting no differences in vision or basal motivation (*P*>0.05). For 3-day spatial hidden-platform test, rats in the fructose-drinking insulin resistance group showed a significant increase in escape latency as compared to those controls (control group and pioglitazone-treated control group; *P*<0.01 for both, Fig. 4B), and the change observed in the fructose-drinking insulin resistance group was partially reversed by administration of pioglitazone (4 trials/day for 3 days, *P*<0.05). As shown in Table 2, in the probe trial, fructose-drinking insulin resistance rats had substantially decreased time of swimming through the hidden escape platform compared with both control rats (5.73±1.34 and 12.1±0.09 respectively, *P*<0.01) and pioglitazone-treated control rats (5.73±1.34 and 12.4±1.21 respectively, *P*<0.01). Pioglitazone-treated fructose-drinking insulin resistance rats had increased times of swimming through the hidden escape platform compared with fructose-drinking insulin resistance rats (9.35±0.45 and 5.73±1.34 respectively, *P*<0.01). Accordingly, fructose-drinking insulin resistance rats showed decreased proportion of path and time through the hidden escape platform, which were in sharp contrast with control and pioglitazone-treated control rats (*P*<0.01), pioglitazone-treated fructose-drinking insulin resistance rats showed increased proportion of path and time compared with fructose-drinking insulin resistance rats (*P*<0.05). Notably, there was no significant difference in all above-mentioned data between control and pioglitazone-treated control groups (*P*>0.05), suggesting that pioglitazone had little or no effect on the cognitive ability of healthy rats. The results indicate that our insulin resistance rat model manifested impaired learning and memory ability which was associated with fructose drinking, and pioglitazone partially improved the cognitive impairments.

**Table 2:** Comparison of learning and memory ability among the groups during the probe trial test.

Groups (n)	The times of swimming through the hidden escape platform	Proportion of path	Proportion of time
**Control (10)**	12.1±0.09	0.37±0.02	0.36±0.12
**Pioglitazone (9)**	12.4±1.21	0.37±0.08	0.36±0.06
**Fructose (8)**	5.73±1.34a	0.20±0.10a	0.16±0.09a
**Pioglitazone+fructose (8)**	9.35±0.45ab	0.31±0.01ac	0.31±0.06ac

Data are shown as mean ± S.E.M.

a *P*<0.01 versus control group;

b *P*<0.01 versus untreated fructose-drinking group;

c *P*<0.05 versus untreated fructose-drinking group.

## Discussion

As a PPARγ agonist and an insulin sensitizer, pioglitazone is widely used for the treatment of T2DM, and has also been reported to be effective in a number of neurodegenerative disease models [19]–[21]. Higher dose of pioglitazone (including 10 mg/kg d) can cross the blood-brain barrier, and pioglitazone can be taken up and detected in the CNS [40], and exerts neuroprotective effect.

Differences in gender have been shown to influence the progression of insulin resistance. Studies using fructose-fed rats have demonstrated that the degree of insulin resistance developed in males is greater than females. Thus, we employed male rats for the present study [41]–[43]. Consistent to literatures [44], [45], we found that fructose had no influence on plasma glucose level. Although fructose does not appear to increase insulin level acutely, chronic exposure seems to cause hyperinsulinemia indirectly [45]. In the present study, we found that high-fructose feeding increased fasting insulin and insulin resistance index in plasma (Table 1), and the insulin resistance is likely mediated by altered activities of the enzymes that regulate hepatic carbohydrates metabolism, such as decreased glucokinase and increased glucose-6-phosphatase activities [46]. Despite the lack of obesity, a fat redistribution is suggested by the observation that enlarged adipose tissue is associated with increased size of adipocyte, which could be in part prevented by metformin [47], [48]. In the present study, 12-week pioglitazone administration did not cause significant changes in weight and plasma glucose level. This is consistent with the data from the 18-month pioglitazone trial in patients with AD carried out by Geldmacher et al [49], in which pioglitazone was found to be well tolerated, without effect on blood glucose, hemoglobin A_1C_, and other hematologic measures.

Oxidative stress is reported as one of the earliest events in the pathogenesis of neurodegenerative diseases including AD [5]. Insulin resistance can result in oxidative stress, leading to irreversible protein aggregation and neuronal degeneration [5]–[7]. In CNS, ROS was removed by antioxidant defense system that works as a complex team. In the present study, all of the three oxidative damage markers: ROS, TBARS and protein carbonyl were found significantly increased in the brains of fructose-drinking insulin resistance rats compared with control rats. SOD converts superoxide to hydrogen peroxide, GPx and CAT convert hydrogen peroxide to water, and they all are primary antioxidant enzymes [38]. GSH, an important non-enzymatic antioxidant, plays a critical role in antioxidant defense in CNS. GSH scavenges ROS by directly reacting with it or may prevent hydrogen peroxide-induced hydroxyl radical formation [17]. In the present study, the activities of SOD, GPx, and CAT were significantly decreased in the brains of fructose-drinking insulin resistance rats. These suggested that the brains in fructose-drinking insulin resistance rats were less efficient to convert hydrogen peroxide to water, and this might lead to the accumulation of hydrogen peroxide. The excessive hydrogen peroxide is also a harmful ROS that can induce cytotoxicity in the brain. Moreover, fructose-drinking insulin resistance rats also showed significant lower levels of GSH and T-AOC in brains. Taken together, it is suggested that insulin resistance induces ROS overproduction, lipid peroxidation, and protein oxidation, as well as decreased anti-oxidative capacity.

PPARγ agonists were used to suppress oxidative damage [7]. Pioglitazone could decrease lipid peroxidation and protein oxidation in the brain [22], [23], and increase the antioxidant capacities in brains of the experimental dementia animal models, cardiomyopathy and nephrotoxicity [23], [50], [51]. In the present study, the markedly increased activities of SOD, CAT and GPx as well as significant higher levels of GSH and T-AOC suggested that pioglitazone administration might reduce the ROS production in fructose-drinking insulin resistance rats. Moreover, the higher T-AOC level also reflected the role of pioglitazone against oxidative stress. Thus, pioglitazone administration strengthens the overall antioxidant defense system.

We found an increased AChE activity and decreased ACh level in fructose-drinking insulin resistance rats in the present study. This is consistent with a previous study that AChE activity was decreased in some of the brain regions in streptozotocin-intracerebroventricularly treated central insulin resistance rats [7]. AChE was over-expressed in response to various oxidative stresses such as free radical and oxidative stress in the brain [17], [52], [53], [54], thus it is possible that the significant increase of AChE activity in the present study is due to oxidative damage induced by insulin resistance. The decreased ACh level in the brains of fructose-drinking insulin resistance rats may simply result from the increased AChE activity. Consistent with previous studies in experimental dementia and scopolamine-induced mice [22], [53], pioglitazone could inhibit AChE activity and restore ACh level in fructose-drinking insulin resistance rats. In our previous studies, we showed that fructose-drinking insulin resistance promotes Aβ-amyloidosis in the brain [25], and AChE activity is increased within and around amyloid plaques [54]. Thus it is possible that the increase of AChE activity and impairment of cognitive function in the present study is due to Aβ induced by insulin resistance.

It is well known that the hippocampus and temporal cortex play important roles in learning and memory. Fructose feeding is used to induce fatty liver disease, and hippocampus-dependent memory function is impaired in this model [3], [4], [45], [55], [56]. Histopathological examination is important for evaluating neuronal damage and drug action [17]. In the present study, we demonstrated that fructose can induce neurodegeneration in brains (Fig. 3). Furthermore, fructose-drinking insulin resistance rats exhibited obviously spatial learning and memory impairments assessed by Morris water maze test (Fig. 4), which supports that long-term fructose drinking impairs cognitive function. However, although pioglitazone cannot ameliorate the irreversible brain damage in fructose-drinking insulin resistance rats, pioglitazone administration indeed rescues learning and memory impairments induced by fructose-drinking, this rescuing effect of pioglitazone on cognition dysfunction is well correlated with its effect on the activities of cholinergic system (AChE and ACh).
